# Contribution of sex and body constitution to three-dimensional lower extremity alignment for healthy, elderly, non-obese humans in a Japanese population

**DOI:** 10.1186/s40634-018-0147-3

**Published:** 2018-08-22

**Authors:** Ryota Katsumi, Tomoharu Mochizuki, Takashi Sato, Koichi Kobayashi, Satoshi Watanabe, Osamu Tanifuji, Naoto Endo

**Affiliations:** 1 Department of Orthopaedic Surgery, Niigata Medical Center, Niigata, Japan; 2 Division of Orthopedic Surgery, Department of Regenerative and Transplant Medicine, Niigata University Graduate School of Medical and Dental Science, Niigata, Japan; 3 Department of Health Sciences, Niigata University School of Medicine, Niigata, Japan

**Keywords:** Healthy elderly human, Lower extremity alignment, Sex, Body constitution

## Abstract

**Background:**

Humans support their bodies exclusively by vertical balance in bipedal locomotion, and the body, especially the lower extremity, generally changes with age. Sex and body constitution are assumed to be associated with lower extremity alignment, but this association remains to be elucidated. This study sought to clarify this association in healthy, elderly, non-obese humans in a Japanese population.

**Methods:**

The present study investigated 55 healthy volunteers (mean age: 70 ± 6 years). A 3D extremity alignment system was applied under weight-bearing conditions on biplane long lower extremities X-rays using a 3D-to-2D image registration technique. The evaluation parameters included 3D hip-knee-ankle angle (3DHKA) alignment in the coronal (coronal alignment) and sagittal planes (sagittal alignment) and rotational alignment between the femur and tibia. The influences of sex and body constitution on all the alignment were analyzed.

**Results:**

Multiple linear regression analysis with the dependent variable of each alignment showed that sex was the dominant factor for coronal and rotational alignment (coronal: *p* <  0.01; rotational: *p* <  0.01), and body weight was the dominant factor for sagittal alignment (*p* <  0.01).

**Conclusions:**

The association of sex with coronal and rotational alignment and of body constitution with sagittal alignment were proved in healthy, elderly, non-obese humans in a Japanese population. This finding can lead to further understanding of the etiology of many diseases and age-related changes.

## Background

Humans support their bodies exclusively by vertical balance in bipedal locomotion (Dubousset, 1994, Skoyles, 2006). The bearing load and forced human vertical balance under gravity produce age-related changes in the spine, pelvis, and lower extremity, leading to a compensatory mechanism and further providing a negative cycle (Dubousset, 1994, Skoyles, 2006, Hasegawa et al., 2016, Ferrero et al., 2016, Jalai et al., 2017).

There are many intrinsic factors affecting the human body that are encoded by genes and extrinsic factors, such as repetitive bearing load and forced vertical balance under gravity. The body constitution, including body weight and height are simple and suitable factors to determine mechanical stress on the human body (Romero-Vargas., 2013, Olchowik et al., 2014, Jalai et al., 2017). The bearing load can be represented by weight, and the balance-retaining ability by the difference in the center of gravity can be simply described by body height (Skoyles, 2006).

Sex is one of the simplest intrinsic factors for expressing the difference in the phenotype due to genes (Bredella., 2017), and sex itself is assumed to markedly affect age-related changes of lower extremity alignment (Ariumi et al., 2010, Mochizuki et al., 2017, Nakano et al., 2016).

Either common assessment of two-dimensional (2D) plain radiographs in the standing position and computed tomography (CT) in the supine position is insufficient for evaluating the deformity and alignment of lower extremities in a three-dimensional (3D) space. Generally, 2D evaluation lacks accuracy and reproducibility, whereas 3D evaluation using CT cannot assess deformity and alignment in weight-bearing (WB) positions. We developed a novel system for 3D assessment of the lower extremity alignment in WB positions, via biplane radiographies using 3D-to-2D image registration techniques (Ariumi et al., 2010, Kobayashi et al., 2009, Mochizuki et al., 2017, Sato et al., 2004, Takagi et al., 2017, Watanabe et al., 2014).

At present, in individuals with normal body constitution, how sex and body constitution affect lower extremity alignment in healthy, elderly, non-obese humans remains controversial. The purpose of this study was to clarify the impact of sex and body constitution on 3D lower extremity alignment in WB positions for healthy, elderly, non-obese humans in a Japanese population, based on the hypothesis that body constitution and sex would provide the different influence on lower extremity alignment in each plane (coronal, sagittal, and axial plane), respectively.

## Methods

First, a total of 62 elderly Japanese subjects who had no knee complaints were prospectively collected. The doctors assessed their general and lower extremities’ conditions by the physical tests and radiographies, and excluded 7 abnormal conditions’ subjects with radiographic knee osteoarthritis (OA). Finally, 55 lower extremities in the right side for the healthy elderly Japanese subjects (26 women, 29 men) were included in this study. The average age and BMI were 70 years (95% CI, 68–71 years) and 21.6 kg/m^2^ (95% CI, 21.1–22.0), respectively. The subjects were not obese and had no history of trauma, pain, knee complaints. They were classified as grades 0–1 based on the Kellgren–Lawrence classification with no radiographic knee OA. Rheumatoid arthritis or other diseases were also excluded.

**Fig. 1:**
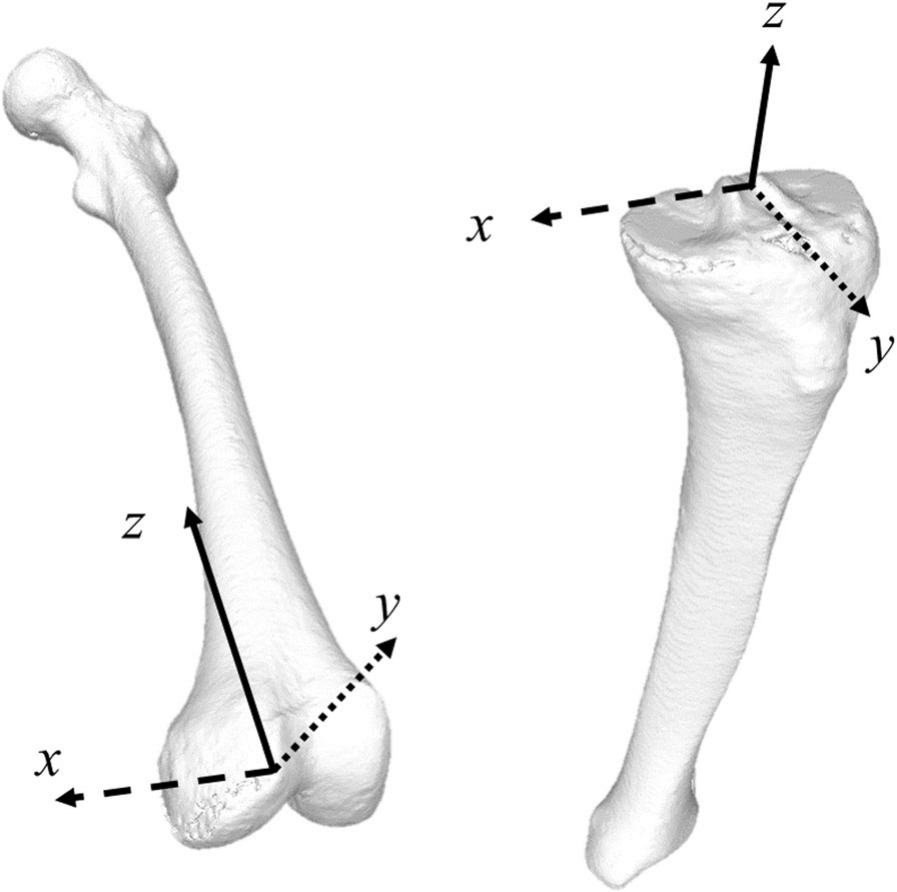
Schematic illustration of femoral and tibial coordinate system

A 3D digital bone model of the femur and tibia of each limb was prepared using CT data and visualization and modeling software (Zedview; LEXI, Tokyo, Japan). The CT data with 1 mm interval in lower extremities were obtained using Canon Aquilion 64 CT (Canon Medical Systems, Tochigi, Japan). The parameters of the scan included a tube voltage of 120 kVp and a tube current of 50–400 mA. For radiation exposure in CT, the mean of CT dose index (CTDI) volume and dose length product (DLP) were 8.33 ± 1.19 mGy and 896.7 ± 129.9 mGy × cm, respectively. Effective dose (ED) was calculated from the DLP using the appropriate body region-specific conversion factor for extremity CT (hip = 7.31, knee = 0.44, ankle = 0.23, μSv/mGy × cm) (Gervaise et al., 2012). The ED values for each body region were summed to calculate the total ED for the study. The mean ED was 7.2 ± 1.0 mSv. The anatomical coordinate systems were installed by a 3D-model digitizing software (Model Viewer; LEXI), applying the anatomical reference points which determined the reference axes (Sato et al., 2004) (Fig. 1).

**Fig. 2:**
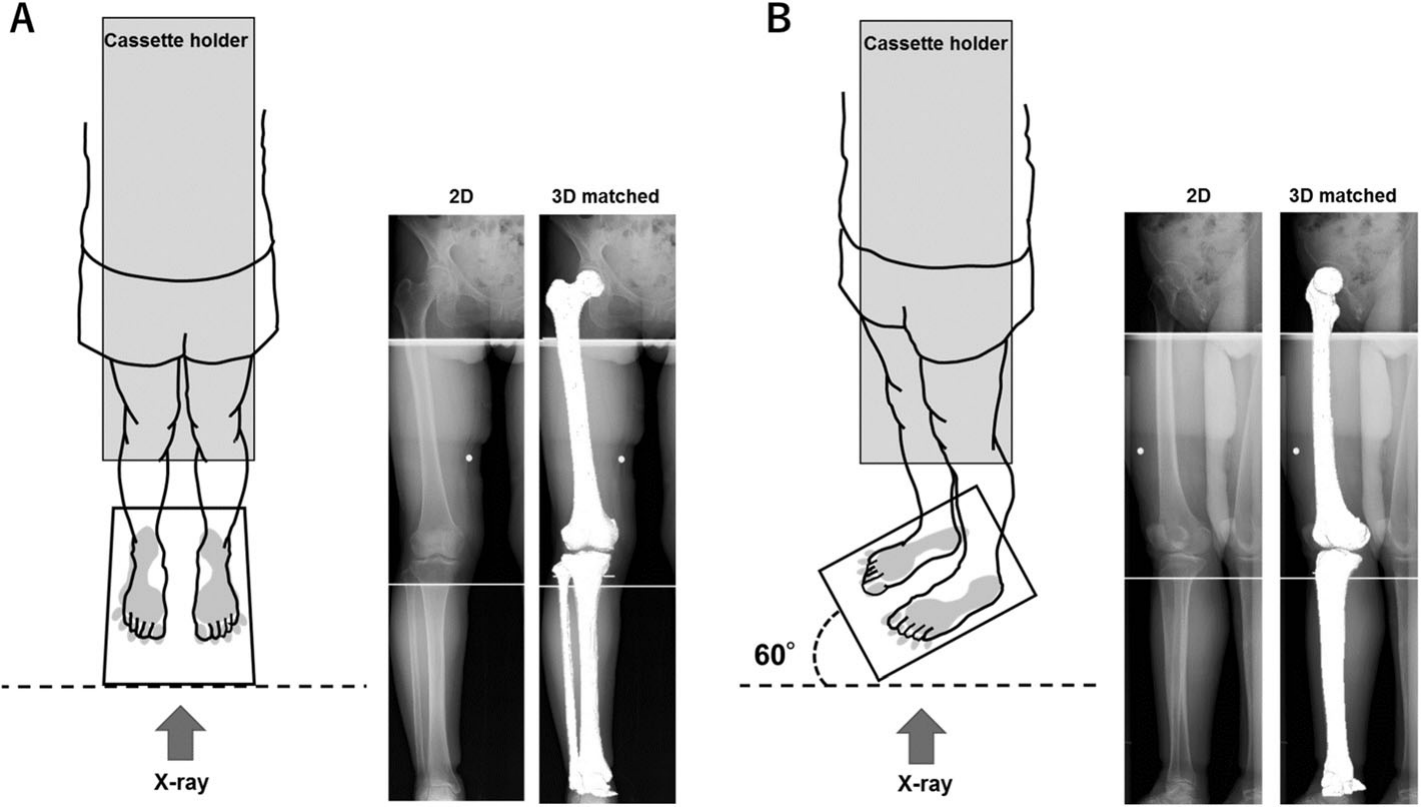
Schematic illustration of the three-dimensional (3D) to two-dimensional (2D) image registration technique. For the biplanar radiography images, the subjects stood at 0° (**a**) and 60° (**b**) to cassette holder. The 3D digital bone models were projected onto the biplanar radiography images in the weight-bearing conditions, using the three-dimensional (3D) to two-dimensional (2D) image registration technique

Biplane computed radiography (CR) images of the lower extremities with 2.58 mGy of radiation exposure were obtained in WB positions, with the knee fully extended and toes in the neutral position, by the 3D lower extremity alignment assessment system (Knee CAS; LEXI, Inc., Tokyo, Japan) (Ariumi et al., 2010, Kobayashi et al., 2009, Mochizuki et al., 2017, Sato et al., 2004, Takagi et al., 2017, Watanabe et al., 2014). The system applied a 3D-to-2D registration technique (Aiumi et al., 2010, Kobayashi et al., 2009, Mochizuki et al., 2013, Mochizuki et al., 2014a, b, Mochizuki et al., 2015, Mochizuki et al., 2016, Mochizuki et al., 2017, Murayama et al., 2016, Sato et al., 2004, Takagi et al., 2017, Tanifuji et al., 2013, Watanabe et al., 2014), and the camera calibration technique (Kobayashi et al., 2009) projected the cited 3D bone models onto the biplane CR images by matching the silhouettes of the 3D models to the contours of the respective CR images. Following these image-matching procedures, the 3D bone model that accurately reproduced the spatial relation between the femur and tibia was displayed (Fig. 2). All alignment parameters were automatically calculated with high accuracy (Kobayashi et al., 2009). The 3D position of each model was recovered by minimizing the difference between the projected outline and contour. The median and maximum values of absolute error in estimating the relative positions for the femur and tibia were within 0.5 mm and 0.61° and 1.6 mm and 1.5°, respectively (Kobayashi et al., 2009).

For the tibial coordinate system, the *z*-axis was determined by a line connecting the midpoint of the tibial eminences to that of the medial and lateral top of the talar dome (positive superiorly). The line perpendicular to the *z*-axis from the mediolateral center of the tibial insertion of the posterior cruciate ligament was defined as the *y*-axis (positive anteriorly). The cross product of the *z*- and *y*-axes was the *x*-axis (positive right). For the femoral coordinate system, the femoral *x*-axis (positive right) was determined by the geometric center axis (a line that connected the centers of the spheres representing the medial and lateral posterior femoral condyles). The midpoint between the centers of these posterior condylar spheres was defined as the origin of the coordinate system. The femoral *y*-axis (positive anteriorly) was a perpendicular line from the origin of the femoral coordinate system to the plane consisting of the geometric center axis and center of the femoral head. The cross product of the *y*- and *x*-axes was the femoral *z*-axis (positive superiorly) (Sato et al., 2004) (Fig. 1).

The evaluation parameters in 3D space, including the 3D hip-knee-ankle (3DHKA) alignment in the coronal (coronal alignment) and sagittal planes (sagittal alignment) and the rotational alignment between the femur and tibia were defined as described below. The influences of sex and body constitution, including body weight, height, and BMI, on these alignment were analyzed.

To evaluate true 3DHKA alignment, the 3D functional axes of the femur (3DFA-f) and tibia (3DFA-t) were defined in a 3D space, and these axes were subsequently projected onto the coronal plane. The 3DFA-f was determined as a line connecting the center of the femoral head and midpoint of the spheres that represented the medial and lateral posterior femoral condyles. The 3DFA-t was determined as a line connecting the midpoint of the eminences of the medial and lateral tibial spines with the center of the ankle joint.

**Fig. 3:**
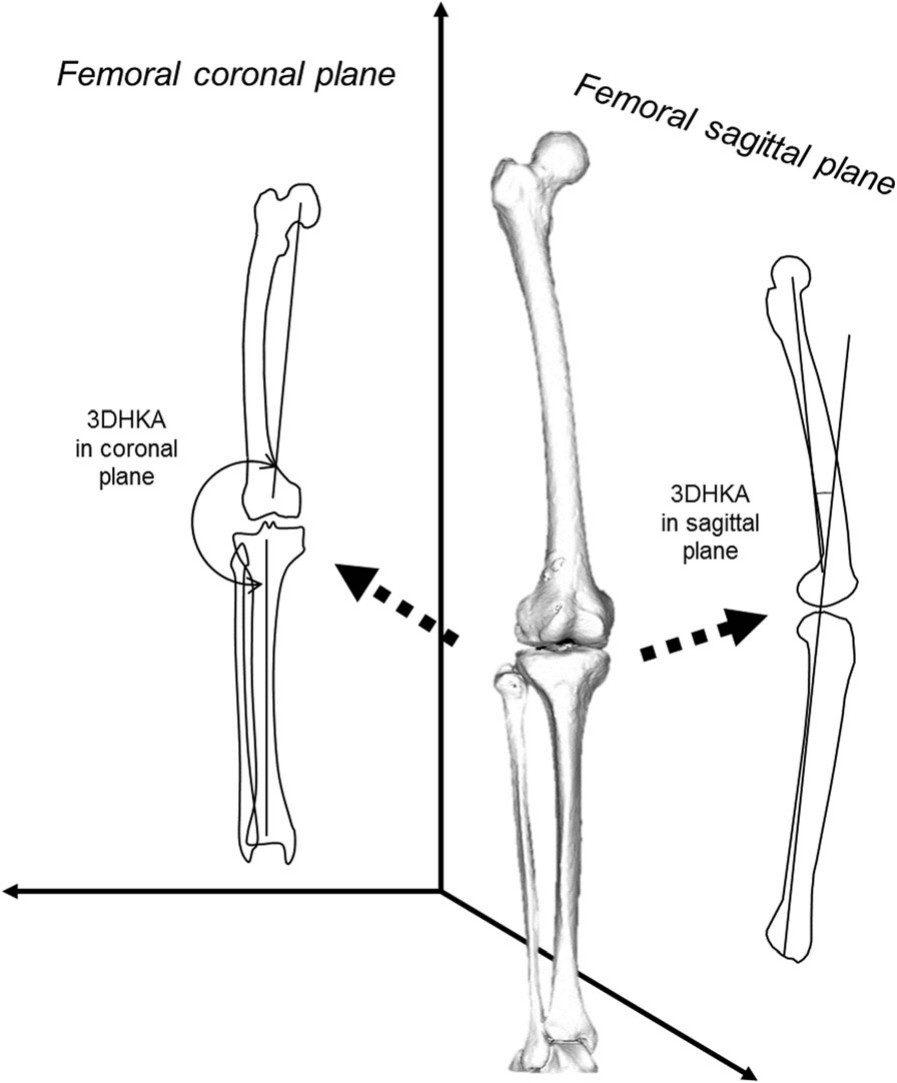
Schematic illustration of coronal and sagittal alignment in a three dimensional space

The angle between 3DFA-f and 3DFA-t projected onto the coronal plane was determined as 3DHKA angle in the coronal plane (coronal alignment) (Ariumi et al., 2010) (Fig. 3). 3DHKA in the coronal plane is more accurate varus–valgus alignment parameter than the common two dimensional HKA. 3DHKA in the coronal plane indicates that larger angles are more varus alignment.

For extension–flexion angle as an alignment, the 3DFA-f and 3DFA-t were projected onto the sagittal plane and defined as 3DHKA angle in the sagittal plane (sagittal alignment) (Ariumi et al., 2010) (Fig. 3). The minus values meant extension angle, which indicated hyperextension of the knee. The common macroscopic knee flexion angle, clinically measured using a goniometer, is essentially the angle between anatomical axes of the femur and tibia. 3DHKA in the sagittal plane shows more extension angle of the knee joint than that measured by the anatomical axes (common knee flexion angle) because the functional axis of the femur has more extension than the anatomical axis relative to the tibia.

**Fig. 4:**
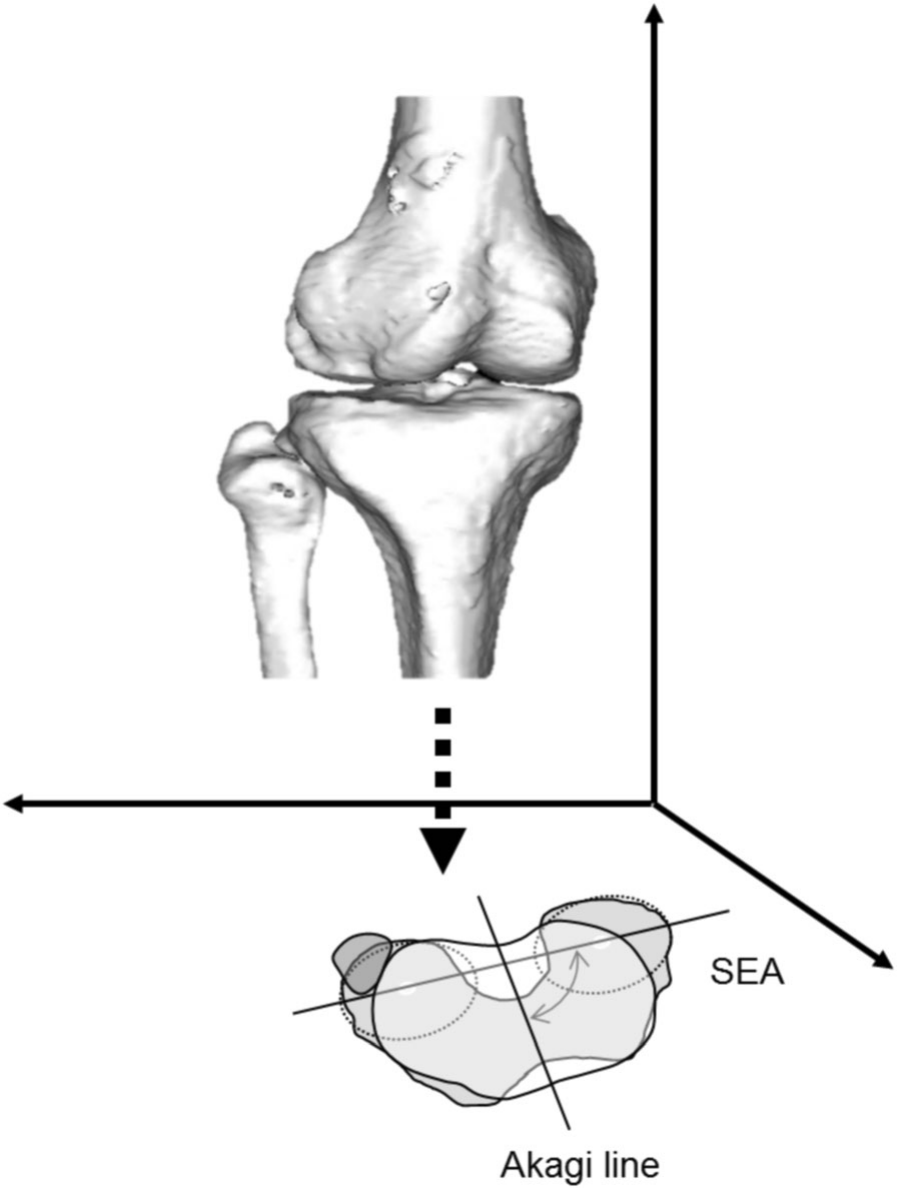
Schematic illustration of rotational alignment in a three dimensional space

The relative rotational alignment between the femur and tibia was defined as the angle between a surgical epicondylar axis and Akagi line projected onto the axial plane in the tibial coordinate system (Fig. 4). Larger values indicated internal rotation of the tibia relative to the femur.

This study was performed according to the protocol approved by the investigational review board of our hospital (IRB number: 2015–01).

### Statistical analyses

Differences in the parameters were statistically analyzed using the Student’s t-test when the data had a normal distribution and equal variances and using the Welch’s t-test when the data had a normal distribution and unequal variances. The Mann–Whitney U test was used when the data did not have a normal distribution. The correlation between alignment and body constitution was evaluated using the Pearson product-moment correlation when the data had a normal distribution and using the Spearman’s rank-order correlation when the data did not have a normal distribution. Multiple linear regression analysis with the stepwise procedure was performed using sex and body constitution as the independent variable and each alignment as the dependent variable. The probability level accepted for statistical significance was set at *p* <  0.05 (SPSS version 21; SPSS, Inc., Chicago, IL, USA).

Regarding the reproducibility of the calculated angles, the intra-observer reproducibility via intra-class correlation coefficient was determined by measuring two times for 52 subjects at the different days. The coronal, sagittal, and rotational alignment were 0.99, 1.00, and 0.94, respectively. The inter-observer reproducibility via inter-class correlation coefficient was determined for 20 subjects by two researchers. The coronal, sagittal, and rotational alignment were 1.00, 0.98, and 0.80, respectively.

The *post-hoc* power analysis showed high power in each parameter when comparing male and females (body weight: power = 1.00, *p* <  0.01; body height: power = 1.00, *p* <  0.01; BMI: power = 1.00, *p* <  0.01; coronal alignment: power = 0.99, *p* <  0.01; sagittal alignment: power = 1.00, *p* <  0.01; rotation alignment: power = 0.95, *p* <  0.01).

## Results

Comparing men and women (Table 1), there were significant differences in all the parameters (*p* <  0.01). In the correlation analysis between alignment and body constitution (Table 2), body constitution was significantly correlated with each alignment in total subjects. Larger weight and higher height showed larger coronal alignment (varus alignment), larger sagittal alignment (knee flexion), and larger rotation alignment (internal rotation of a tibia to a femur) (Table 2).

**Table 1:** Differences in each parameter between men and women

Variable	Men		Women		
	mean	95%CI	mean	95%CI	*p* value
Age (years)	71	69–73	68	66–70	0.03*
Body weight (kg)	63.7	61.0–66.5	48.3	46.3–50.3	< 0.01*
Body height (cm)	166.3	164.6–168.0	155.4	153.3–157.5	< 0.01*
Body mass index (BMI) (kg/m^2^)	23.0	22.2–23.8	20.0	19.3–20.6	< 0.01*
HKA in coronal plane (°)	182.7	181.9–183.6	180.6	179.7–181.5	< 0.01*
HKA in sagittal plane (°)	0.4	−2.6 – 3.1	−4.4	−6.0 – − 2.7	< 0.01*
Rotational alignment (°)	90.0	87.2–92.7	84.7	82.0–87.3	< 0.01*

*95%CI* 95% confidence interval, *HKA* hip-knee-ankle angle; HKA in sagittal plane: the minus values mean extension angle; Rotational alignment: the larger values mean internal rotation of the tibia relative to the femur; *Significant difference = *p* <  0.05

**Table 2:** Correlation between body constitution and alignment

	HKA in coronal plane		HKA in sagittal plane		Rotation	
Total (males and females)	CC	*p* value	CC	*p* value	CC	*p* value
Body weight	0.37	< 0.01*	0.46	< 0.01*	0.34	< 0.01*
Body height	0.41	< 0.01*	0.31	< 0.01*	0.34	< 0.01*
Body mass index (BMI)	0.28	0.02*	0.45	< 0.01*	0.27	0.02*
Men						
Body weight	0.02	0.46	0.45	< 0.01*	0.20	0.15
Body height	0.12	0.28	0.18	0.18	0.14	0.23
Body mass index (BMI)	−0.04	0.43	0.46	< 0.01*	0.17	0.18
Women						
Body weight	0.12	0.27	−0.23	0.13	−0.07	0.38
Body height	0.15	0.24	−0.20	0.17	0.07	0.37
Body mass index (BMI)	0.05	0.40	−0.13	0.26	−0.14	0.26

*Significant difference = *p* <  0.05

**Fig. 5:**
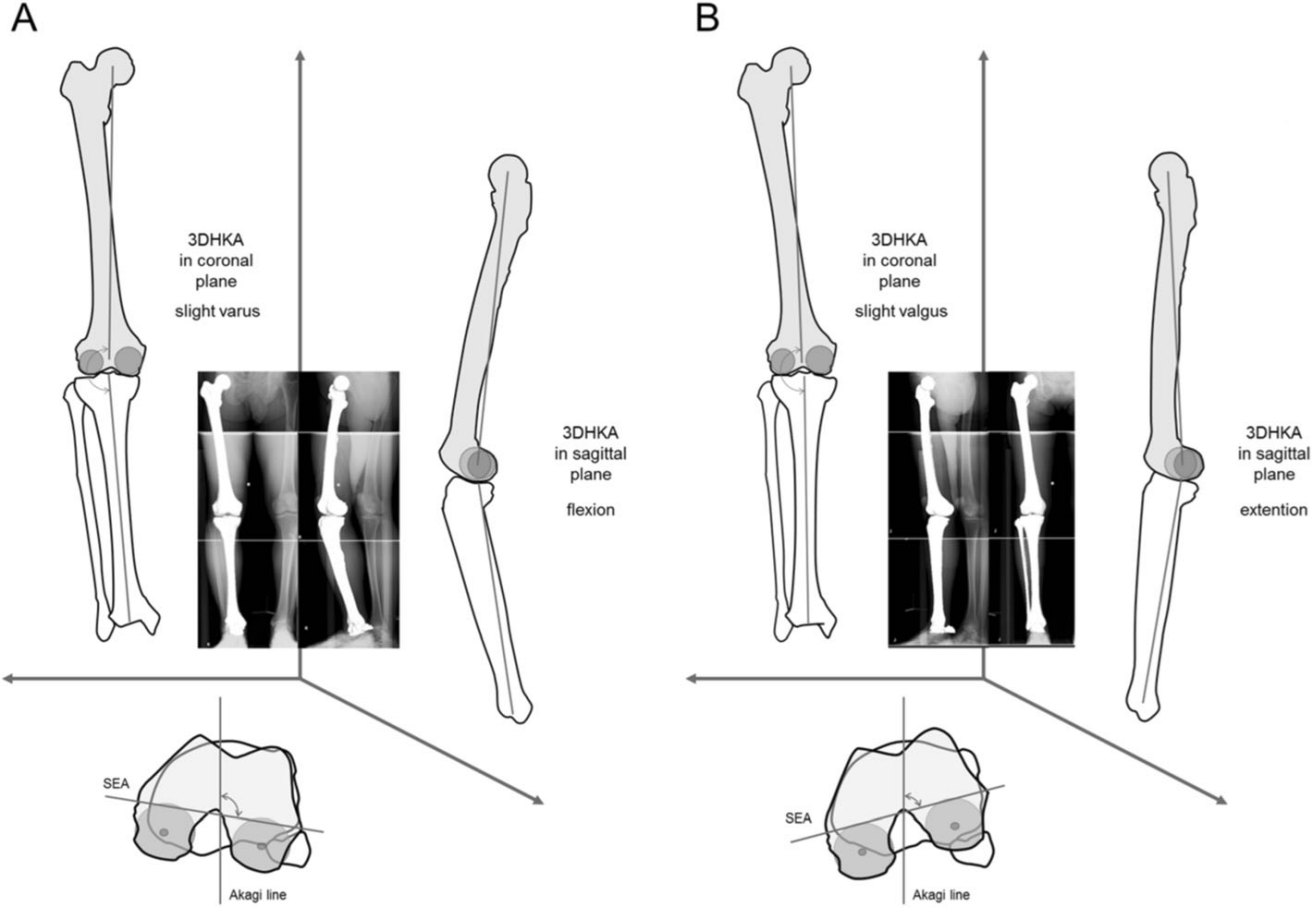
Representative images showing the difference between men (**a**) and women (**b**)

Multiple linear regression analysis with the dependent variable of each alignment showed that sex had the strongest influence on coronal (women – valgus alignment, *p* <  0.01) and rotational alignment (men – internal rotation of a tibia to a femur, *p* <  0.01) and body weight had the strongest influence on sagittal alignment (larger weight – more knee flexion, *p* <  0.01) (Table 3). The representative images showing the difference between men and women were demonstrated in Fig. 5.

**Table 3:** Multiple linear regression analysis with dependent variable of alignment

Dependent variable	Selective variable by stepwise procedure	Beta	*t* value	*p* value
HKA in coronal plane	Sex	−0.38	− 4.29	< 0.01*
HKA in sagittal plane	Body weight	0.48	5.70	< 0.01*
Rotation	Sex	−0.33	−3.65	< 0.01*

Definition of sex variable, men = 0, women = 1; *Significant difference = *p* < 0.05

## Discussion

The most important findings were that sex was the dominant factor for coronal and rotational alignment and that body constitution was the dominant factor for sagittal alignment in the standing position for healthy, elderly, non-obese humans in a Japanese population. For clinical relevance, these associations can lead to further understanding of the etiology of many diseases and age-related changes.

This study proved a greater contribution of sex to coronal alignment (women – more valgus alignment). Young and middle-aged men have reported to be more varus alignment than that in women (Nakano et al., 2016). Women have a wider pelvis to give birth (Gruss LT et al., 2015). The wider pelvis possibly leads to the compensatory mechanisms to align the center of gravity. That may be why women have higher anteversion of the femoral neck in the hip joint (Mochizuki et al., 2017) and more valgus alignment of the lower extremity than those in men (Nakano et al., 2016).

The sex difference in sagittal alignment was demonstrated in the present study. Men had more knee flexion than women under the standing position. The importance of knee flexion is well described as a compensatory mechanism for maintaining horizontal gaze. Although there are many compensatory mechanisms involving the pelvis and lower extremities, the primary aim of these efforts is to maintain the center of gravity centrally positioned over the feet (Jalai et al., 2017) and to preserve a horizontal gaze (Hasegawa et al., 2016). Several studies examined knee flexion in relation to the lack of lumbar lordosis and showed that the femoral angle was significantly associated with pelvic tilt, and that knee flexion was preferentially adopted (Lee CS et al., 2013). The sex-related difference in knee flexion in this study may have been caused due to complicated connections among the compensatory mechanisms for spinopelvic deformity, body constitution, neurological factors, bony morphology such as tibial posterior tilt, and other factors.

Focusing on the effect of body constitution on sagittal alignment, body weight was associated with sagittal alignment. Previous research demonstrated the influences of the compensatory mechanism of the pelvis and lower extremities by WB load (Romero-Vargas., 2013, Jalai et al., 2017). Excessive WB in proximity to the hip and pelvis causing global anterior shift forces the predominant recruitment of supplemental lower limb compensation to regulate the sagittal offset (Jalai et al., 2017). As the latest finding, our study proved that body weight, even when the BMI is within the normal limit, is the dominant factor for flexion alignment.

In addition to body weight, the present study demonstrated the association of body height with sagittal alignment. The center of gravity in the human body is usually high due to human lower extremity constituting a large percentage of the total height (Skoyles, 2006). The risk of falling is linked to the height of the center of gravity above the ground and area of its support base, which markedly increases the role of balancing skills (Skoyles, 2006). For the compensatory mechanism to maintain balance, taller subjects, especially men, may unconsciously require more knee flexion under the standing position.

Rotational alignment was associated with sex. Kinematics and bony morphology can explain the influence of sex on rotational alignment. Knee kinematics include tibial internal rotations to a femur as knee flexion (Mochizuki et al., 2013, Mochizuki et al., 2014a, b, Mochizuki et al., 2015, Murayama et al., 2016, Tanifuji et al., 2013), especially the drastic screw-home rotational motion which occurs around knee extension. The difference of 5° in sagittal alignment between men and women can provide the difference in rotation to a certain degree, which partially explained the sex difference of rotational alignment. Concerning bony morphology, the sex difference of the femoral neck anteversion, as the rotational factor, has been reported (Mochizuki et al., 2017). The contribution of sex to rotational alignment may be due to the combination of kinematics, bony morphology, or other factors.

There are several limitations in this study. First, the sample size was not large, but the statistical power of this study was sufficient. Second, the average age was different between men and women, but the difference between the two was approximately 3 years, which was minimal for elderly participants and may not affect the study results. Third, the spine alignment that assumingly had influences on the lower extremity alignment was not assessed. Although we were interested in the association, the system could not evaluate the whole body including spine alignment. The novel system will be necessary in order to accurately investigate the association between spine and lower extremity alignment. Fourth, this study had radiation exposure of CT. For accurate assessment of bony deformity and alignment, CT is the best modality and then applied in this method. The radiation exposure was the same degree in the common clinical setting and did not directly cause adverse effect for the participants. Fifth, this study was conducted for only Japanese population and the difference in races remains unclear.

## Conclusions

This study proved that sex was the dominant factor affecting coronal and rotational alignment, whereas body constitution was the dominant factor affecting sagittal alignment in the standing position for healthy, elderly, non-obese humans in a Japanese population.

## Abbreviations

2D: two-dimensional
3D: three-dimensional
BMI: body mass index
CR: computed radiography
HKA: hip-knee-ankle angle
